# Reversible Parkinsonism caused by Influenza B‐associated encephalitis affecting bilateral basal ganglia: A case report

**DOI:** 10.1111/cns.13278

**Published:** 2019-12-10

**Authors:** Yue‐Li Zhu, Xiao‐Ming Guo, Zun‐Bo Qin, Zhi‐Jian Zhou, Jin Cao, Ji‐Min Wu, Jia‐Li Pu

**Affiliations:** ^1^ Department of Neurology Second Affiliated Hospital School of Medicine Zhejiang University Hangzhou China; ^2^ Department of Neurosurgery Second Affiliated Hospital School of Medicine Zhejiang University Hangzhou China; ^3^ Department of Neurology Hengdian Wenrong Hospital Jinhua China; ^4^ Department of Neurology Affiliated Shaoxing Hospital of Traditional Chinese Medicine Zhejiang Chinese Medical University Shaoxing China

**Keywords:** basal ganglia, encephalitis, influenza B virus, mechanism, Parkinsonism

Dear Editor

Influenza viruses are important human pathogens that often cause respiratory tract infections, while neurological complications are rare.^1^ Secondary Parkinsonism following viral infections usually occur several years after encephalitis, but rarely during the acute encephalitic phase.^2^ A range of viruses are associated with acute and chronic Parkinsonism, such as the influenza virus, coxsackievirus, and human immunodeficiency virus (HIV).^2^ To our knowledge, Parkinsonism resulting from the influenza B virus has never been reported. Here, we describe an unusual encephalitis case, in which acute reversible Parkinsonism occurred when a influenza B virus infection affected the bilateral basal ganglia.

An unemployed 50‐year‐old man was admitted to our hospital complaining of progressively slower movement and a gait disturbance for half a month. Three weeks prior, he initially had a 38°C fever, sore throat, cough, and expectoration. A nasopharyngeal swab tested in another hospital showed the antigen and nucleic acid of the influenza B virus were positive. As the diagnosis was flu, anti‐infective drugs were used, including oseltamivir and cefuroxime. His symptoms were in remission one week later, but he developed progressively slower movement and a gait dysfunction. After being admitted to our hospital, he felt fatigued and was dull in response, being unable to do simple arithmetic. A neurological examination showed generalized bradykinesia (Video S1), and an intermittent resting tremor of the bilateral upper limbs was noted, as well as hypomimia. The modified Medical Research Council score for muscle strength was grade 4+ in all limbs. He had a history of kidney transplantation for 6 years, with immunosuppressant treatment with enteric‐coated mycophenolate sodium and tacrolimus. And he denied hypoxia and toxin exposure history. The patient explicitly agreed to his inclusion in this case report and gave written informed consent for publication.

Serological tests including full blood counts, blood glucose, thyroid function, liver and renal function, and tumor markers did not show any abnormity. Metabolic as well as endocrine dysfunctions were ruled out as the primary causes. A lumbar puncture was performed on the second day after admission, which found cerebrospinal fluid (CSF) pressure was normal (15 cm H_2_O), but an elevated protein concentration of 48.2 mg/dL was detected. Tests for antibodies mediating autoimmune encephalitis (anti‐NMDAR, ‐AMPAR, ‐LGI1, ‐CASPR2, ‐GABA_B_R, ‐DPPX, ‐IgLON5, ‐GAD65) in CSF and paraneoplastic syndromes (anti‐Hu, ‐Yo, ‐Ri, ‐CV2, ‐PNMA2, ‐Amphiphysin) in serum were all negative. The test for influenza B viral RNA from the CSF was negative. Brain magnetic resonance imaging (MRI) revealed hypointensity in the bilateral caudate head and putamen on T1‐weighted sequences (Figure 1A) and hyperintensity on T2‐weighted sequences (Figure 1B) and fluid‐attenuated inversion recovery sequences (Figure 1C).

**Figure 1:**
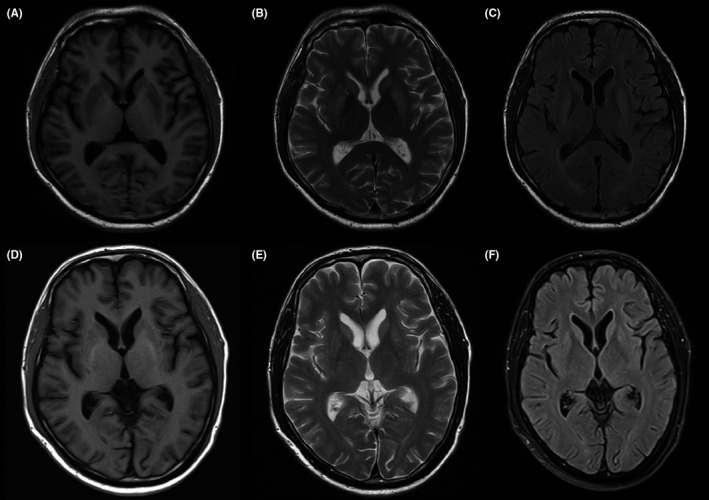
Axial MRI images at admission show lesions in bilateral caudate head and putamen: (A) hypointensity on T1‐weighted images, (B) hyperintensity on T2‐weighted images, (C) hyperintensity on T2‐weighted fluid‐attenuated inversion recovery sequences. Repeated MRI images 3 mo later show lesions with obvious improvement in bilateral caudate head and putamen: (D) hypointensity on T1‐weighted images, (E) hyperintensity on T2‐weighted images, (F) hyperintensity on T2‐weighted fluid‐attenuated inversion recovery sequences

The patient was diagnosed with encephalitis of the basal ganglia resulting from influenza B virus, and treated with intravenous methylprednisolone (80 mg/d for 8 days), madopar (each containing 200 mg levodopa and 50 mg benserazide) (62.5 mg TID), and amantadine (0.05 g BID). After one week of treatment, a significant improvement in his movement was observed, especially his gait (video). Then, the patient was discharged. He stopped taking madopar and amantadine one month later. A 3‐month follow‐up brain MRI showed the abnormal signal in bilateral basal ganglia had markedly decreased (Figure 1D‐F). And the patient returned for 5‐month follow‐up after discharge, his slow response and movement further improved and his facial expressions were more varied than before.

Several sporadic cases of postencephalitic Parkinsonism have been reported since the pandemic of 1917‐1928.^3^ The viruses involved in these cases include coxsackievirus, HIV, Epstein‐Barr virus, western equine virus, influenza A virus, and West Nile virus.^2^, ^3^ A viral infection with substantia nigra involvement leading to secondary Parkinsonism has been reported previously.^4^ However, a virus affecting the bilateral basal ganglia that results in Parkinsonism is uncommon. A review revealed that MRI findings of bilateral signal abnormalities in the basal ganglia and thalami can be seen in West Nile encephalitis with associated Parkinsonism.^5^ The pathophysiology of the basal ganglia is still unclear and here are some hypotheses to explain the phenomenon. Yoshii et al reported that the basal ganglia are vulnerable to infections carried via the blood, as they are richly vascularized with an end‐vessel vascular supply.^6^ In addition, Jang et al confirmed the influenza virus can enter the central nervous system via the olfactory, trigeminal, and vagus nerves.^2^ The patient initially had oropharyngeal symptoms, which may have made him vulnerable to encephalic infections. Finally, autoimmunity could be an underlying mechanism, since it has been reported that infectious agents can trigger autoimmune encephalitis.^7^ The patient had an immune dysfunction because of consecutive immunosuppressant treatments, which could have been part of the pathogenesis.

Virological analyses are important for the diagnosis of neurological complications of influenza. Interestingly, in the current case, the test for influenza B viral RNA from the CSF was negative. Positive virus isolation from the CSF is rarely reported, perhaps because CSF contains low amounts of virus or the viruses have disappeared by the time of sampling.^8^ However, influenza virus RNA fragments in CSF have been detected in cases with a rapid onset of encephalitis after 1‐2 days of influenza infection.^8^ In our case, lumbar puncture was performed three weeks after influenza infection. It might cause the failure of detection of influenza virus B‐specific RNA fragments in the CSF. And we should note that virus isolation from nasopharyngeal aspirates is also valuable.

Tacrolimus‐induced Parkinsonism should be considered in the differential diagnosis. Cytostatic treatment after solid organ transplantation could lead to symptoms of Parkinsonism which is more commonly seen under tacrolimus treatment.^9^, ^10^ However, to our knowledge, Parkinsonism often occurs in the first several days of immuno‐suppressive treatment.^9^, ^10^ And improvement of clinical symptoms will be noted after the discontinuation of cytostatic treatment.^9^ Since our patient had a 6‐year history of cytostatic treatment and his symptoms of Parkinsonism improved without withdrawal of the drugs, then this diagnosis was excluded.

With regard to clinical features of this disease, influenza‐like symptoms, including acute onset of fever and respiratory symptoms usually occur first, and can rapidly progress to Parkinsonism, manifested as bradykinesia, static tremor, rigidity, and hypomimia. The combination of levodopa and amantadine is effective for these Parkinsonism symptoms because amantadine targets the M2 protein channel on the surface of the influenza virus.^2^

In conclusion, we report a rare case of reversible Parkinsonism caused by the influenza B virus producing lesions of the basal ganglia, but the mechanism of basal ganglia involvement remains hypothetical. We hypothesize an interaction between viral infection and immune factors may have played an important role in the pathogenesis. The exact pathophysiology warrants investigation.

## CONFLICT OF INTEREST

The authors declare no conflict of interest.
